# Help the Hospitals

**Published:** 1886-12-18

**Authors:** 


### HELP THE HOSPITALS.

Oh! help! the poor are poorer still,
When sickness finds their dwelling;
Then help with right good heart and will,
For hearts with grief are swelling.

Oh! help! there are many fathers low,
Whose hearts are filled with sorrow,
Who think with a sad and gathering brow,
Of the dark and gloomy morrow.

Oh! help! the poor man's source of wealth
Is British pluck and sinew;
Then help to give him back his health,
As much as lieth in you.

Oh! help! a thousand voices cry,
With hearts well-nigh desponding,
And God, the mighty God on high,
Will bless thy quick responding.

